# Functionalized Silver Nanoparticles as Multifunctional Agents Against Gut Microbiota Imbalance and Inflammation

**DOI:** 10.3390/nano15110815

**Published:** 2025-05-28

**Authors:** Mihaela Stoyanova, Vera Gledacheva, Miglena Milusheva, Mina Todorova, Nikoleta Kircheva, Silvia Angelova, Iliyana Stefanova, Mina Pencheva, Yulian Tumbarski, Bela Vasileva, Kamelia Hristova-Panusheva, Zlatina Gospodinova, Natalia Krasteva, George Miloshev, Milena Georgieva, Stoyanka Nikolova

**Affiliations:** 1 Department of Organic Chemistry, Faculty of Chemistry, University of Plovdiv, 4000 Plovdiv, Bulgaria; stoyanova@uni-plovdiv.bg (M.S.); miglena.milusheva@uni-plovdiv.bg (M.M.); minatodorova@uni-plovdiv.bg (M.T.); tanya@uni-plovdiv.bg (S.N.); 2 Department of Medical Physics and Biophysics, Faculty of Pharmacy, Medical University of Plovdiv, 4002 Plovdiv, Bulgaria; iliyana.stefanova@mu-plovdiv.bg (I.S.); mina.pencheva@mu-plovdiv.bg (M.P.); 3 Department of Bioorganic Chemistry, Faculty of Pharmacy, Medical University of Plovdiv, 4002 Plovdiv, Bulgaria; 4 Institute of Optical Materials and Technologies “Acad. J. Malinowski”, Bulgarian Academy of Sciences, 1113 Sofia, Bulgaria; nkircheva@iomt.bas.bg (N.K.); sea@iomt.bas.bg (S.A.); 5 University of Chemical Technology and Metallurgy, 1756 Sofia, Bulgaria; 6 Department of Microbiology and Biotechnology, Technological Faculty, University of Food Technologies, 4002 Plovdiv, Bulgaria; tumbarski@abv.bg; 7 Laboratory of Molecular Genetics, Epigenetics and Longevity, Institute of Molecular Biology “R. Tsanev”, Bulgarian Academy of Sciences, 1113 Sofia, Bulgaria; byvasileva@gmail.com (B.V.); karamolbiol@gmail.com (G.M.); milenageorgy@gmail.com (M.G.); 8 Institute of Biophysics and Biomedical Engineering, Bulgarian Academy of Sciences, 1113 Sofia, Bulgaria; kameliahristova@abv.bg (K.H.-P.); natalia.krasteva@yahoo.com (N.K.); 9 Institute of Plant Physiology and Genetics, Bulgarian Academy of Sciences, Acad. G. Bonchev Str. Bl.21, 1113 Sofia, Bulgaria; zlatina.go@abv.bg

**Keywords:** antimicrobial, antifungal, silver nanoparticles, DFT, spasmolytic, anti-inflammatory activity

## Abstract

Human pathogenic fungi are the source of various illnesses, including invasive, cutaneous, and mucosal infections. One promising solution is using nanoparticles (NPs) as an antifungal agent. The current study aims to assess the antimicrobial and antifungal effects of drug-loaded silver nanoparticles (AgNPs) with previously reported mebeverine analogue (MA) as a potential drug candidate targeting gut microbiota and inflammation in the gastrointestinal tract. Density Functional Theory (DFT) calculations were conducted to identify possible mechanisms by which AgNPs could prevent microorganisms from growing. In vitro and ex vivo anti-inflammatory, in vitro antimicrobial, ex vivo spasmolytic activities, and in vitro hepatic cell morphology and proliferation of drug-loaded AgNPs were assessed. The drug-loaded AgNPs were considered to have promising antifungal activity against all tested fungal strains, *Aspergillus niger*, *Penicillium chrysogenum*, and *Fusarium moniliforme*, and yeasts, *Candida albicans*, *Saccharomyces cerevisiae*, and good antimicrobial activity against Gram-positive and Gram-negative bacterial strains. The results of in vitro and ex vivo determination of anti-inflammatory activity indicated that the drug-loaded AgNPs preserved MA’s anti-inflammatory activity and decreased inflammation. A similar effect was observed in spasmolytic activity measurements. Drug-loaded AgNPs also influenced the morphology and proliferation of hepatic cells, indicating a potential for improved gut and liver therapeutic efficacy. Each test was performed in triplicate, and the results were reported as mean values. Based on the results, drug-loaded AgNPs might be a promising antimicrobial agent, maintaining the MA’s potential as a spasmolytic and anti-inflammatory agent. Future in vivo and preclinical experiments will contribute to establishing the in vivo properties of drug-loaded AgNPs.

## 1. Introduction

The gut microbiome is a complex and dynamic ecosystem that constantly contains bacteria, viruses, fungi, and archaea in the gastrointestinal (GI) tract and a protective epithelial barrier [1]. These microorganisms form a complex micro-ecosystem influencing the host’s physiology [2]. For example, the commensal gut microbiota supports host metabolism [3], preserves intestinal barrier function [4], and regulates the immune system, among other functions [5]. The gut’s fungi, bacteria, and viruses have a stable but dynamic connection with each other under physiological settings. This association might be hostile, synergistic, or neutral [6]. The common fungus *Candida albicans*, for instance, interacts with a variety of bacteria, such as *Clostridium difficile* and *Enterococcus faecalis* [7,8], changing their assembly and function through interactions with cell membranes, competition or cooperation for nutrients, and the generation of antimicrobial peptides and secondary metabolites [9,10,11]. A delicate balance between defence against enteric pathogens and tolerance to commensal microbes must be struck to maintain intestinal homeostasis. Inflammatory illnesses of the GI tract, including inflammatory bowel disease (IBD), have been linked to a disruption in this balance [12]. Many variables can influence these interactions and mold the gut microbiota, leading to either stability or instability [13,14,15,16].

Fungal illnesses cause about 1.6 million deaths annually, which is comparable to tuberculosis and more than three times the estimated number of deaths from malaria [17]. Immunocompromised people are more susceptible to fungal infections than healthy people, which explains this. Furthermore, the number of immunocompromised individuals has increased due to the HIV epidemic and medical advancements over the past few decades (such as the discovery of antibiotics, improvements in cancer treatment, and surgical transplants), which has caused fungal infections to change from being a rare cause of disease to a significant contributor to human morbidity and mortality globally [18].

Human pathogenic fungi are the source of various illnesses, including invasive, cutaneous, and mucosal infections [19]. The cause of death and morbidity, particularly in those with weakened immune systems, is invasive and potentially fatal fungal infections. There are now fewer treatment medicines available to treat invasive fungal infections than there are for bacterial infections [20]. The limited number of antifungal drugs that are now accessible, antifungal resistance, and the increased toxicity of these treatments are also associated with the increased mortality and morbidity brought on by fungal infections [21,22,23].

One promising solution is using NPs as an antifungal agent [24,25]. Metallic NPs have already been shown to be an efficient and different approach to fungicidal agents [26,27]. NPs have enormous promise due to their inherent antifungal qualities and capacity to transport antifungal medications [28,29]. For example, gold NPs can disrupt mitochondrial calcium homeostasis, which causes gold NP-mediated *Candida albicans* cell death [27,30]. Furthermore, copper and copper oxide NPs demonstrated notable anti-pathogenic fungal effects [31]. Iron oxide NPs showed strong antifungal properties against *P. chrysogenum* and *A. Niger* [27,29]. Moreover, zinc oxide NPs have been shown to reduce fungi’s growth dramatically [32,33]. These NPs have already demonstrated strong antifungal effects against various harmful fungal species, such as *Aspergillus*, *Fusarium*, *Candida*, and numerous others [34,35]. Research and development of antifungal NPs are now conducted using a variety of approaches, such as chemical modification of NPs and combination with currently available medications.

When applied to many harmful fungi, AgNPs demonstrated potent antifungal effects. Humanity has long recognized silver’s antibacterial properties in addition to its status as one of the seven metals of antiquity. Due to its more significant toxicity to germs and far lower toxicity to mammalian cells, silver has regained attention due to the recent growth of multidrug-resistant bacteria and the need for new approaches [36,37]. Therefore, due to their demonstrated antifungal efficacy, there is increasing interest in using AgNPs to treat fungal infections.

Recently, we synthesized AgNPs functionalized with two spasmolytics—mebeverine (MBH) and previously synthesized 2-amino-N-(1-(3,4-dimethoxyphenyl)propan-2-yl)benzamide (mebeverine analogue, MA)—and evaluated their cytotoxic and genotoxic effects [38]. We found that the synthesized AgNPs show significant promise as drug delivery vehicles, enhancing bioavailability and efficacy. Their ability for targeted administration and low genotoxicity indicates that they may be developed into safe and effective medicines.

**Figure 1:**
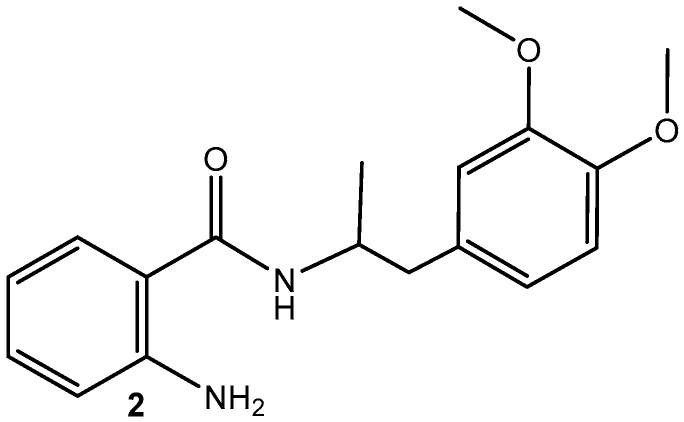
Structure of MA (**2**).

The current study aims to assess the antimicrobial and antifungal effects of drug-loaded AgNPs (**1**) with MA(**2**) (Figure 1).

In silico methods using Density Functional Theory (DFT) were used to provide evidence at a molecular level for a thermodynamically justified antifungal and antimicrobial effect of drug-loaded AgNPs. Previously, the spasmolytic activity and anti-inflammatory potential of MA were reported [39]. Therefore, it was essential to investigate these activities concerning MA’s deposition on AgNPs [40]. Developing synthesis and functionalization strategies that guarantee effectiveness, safety, and biocompatibility is crucial to investigate the possible biomedical uses of silver nanoparticles (AgNPs) [41,42]. AgNP risk evaluations examine biological consequences, possible origins, and effective methods for reducing adverse effects [43,44]. It was, therefore, essential to evaluate the morphology and proliferation of hepatic cells for drug-loaded AgNPs. Our understanding of AgNPs will be improved through advancements in experimental and computational methods, thereby facilitating their secure and practical use across various domains [45].

A systematic study investigated the MA-loaded silver nanoparticles in their entire capacity. The presented results are based on in silico (DFT), in vitro (antimicrobial, inhibition of albumin’s denaturation), and ex vivo (anti-inflammatory and spasmolytic) methodologies. This combined approach allows for drawing a clearer picture of silver nanoparticles’ potential to target gut microbiota and inflammation in the gastrointestinal tract.

## 2. Materials and Methods

### 2.1. Synthesis of MA (2) (Scheme 1) [39]

**Scheme 1:**
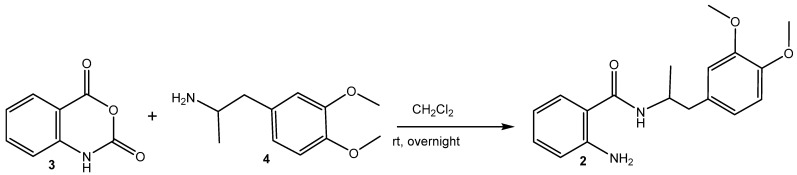
Reaction pathway for the synthesis of MA (**2**).

A mixture of isatoic anhydride (**3**) (1.63 g, 10 mmol) and 1-(3,4-dimethoxyphenyl)propan-2-amine (**4**) (initially prepared from 1-(3,4-dimethoxyphenyl)propan-2-one) [46,47] (2.93 g, 15 mmol) dissolved in dichloromethane (30 mL) was stirred overnight at rt. The resulting solution was filtered on neutral Al_2_O_3_ and concentrated (Scheme 1).

Spectral data confirmed the structure of the obtained 2-amino-N-(1-(3,4-dimethoxyphenyl)propan-2-yl)benzamide (MA, **2**): m.p. 152.8 *◦*C, ^1^H NMR (500 MHz, CDCl_3_) δ 1.14 (d, *J* = 6.7, 3H, CH_3_), 2.70–2.80 (m, 2H, CH_2_), 3.75 (s, 3H, OCH_3_) 3.78 (s, 3H, OCH_3_), 4.32 (dq, *J* = 13.4, 6.7 Hz, 1H, CH), 5.51 (broad s, 1H, NH_2_), 5.86 (d, *J* = 7.9 Hz, 1H, NH), 6.56–6.59 (m, 1H, Ar), 6.66–6.68 (m, 3H, Ar), 6.74 (d, *J* = 5, 1H, Ar), 7.11–7.15 (m, 2H, Ar); ^13^C NMR (126 MHz, CDCl_3_) δ 168.49, 148.86, 147.72, 147.46, 132.25, 130.37, 126.98, 121.59, 117.91, 117.43, 117.07, 112.62, 111.18, 55.91, 55.84, 46.16, 41.66, 19.99; FT-IR, cm^−1^: 3467 ν_as_ (-N-H, -NH_2_), 3371 ν_s_ (-N-H, -NH_2_), 3283 ν (-N-H, >NH-amide), 3067, 3055, 3033 ν (Csp^2^-H, -Ph), 2998 ν_as_ (Csp^3^-H, -OCH_3_), 2972 ν_as_ (Csp^3^-H, -CH_3_), 2935, 2914 ν_as_ (Csp^3^-H, -CH_2_-), 2874 ν_s_ (Csp^3^-H, -CH_3_), 2838 ν_s_ (Csp^3^-H, -OCH_3_), ν_s_ (Csp^3^-H, -CH_2_-), 1626 ν (>C=O), secondary amide I, 1584 ν (C-C=C, -Ph1,2,4), 1539, 1514 δ(N-H) and ν (C-N), trans-secondary amide II, 1463 ν (C-C=C, -Ph_ortho_), ν (C-C=C, -Ph1,2,4), δ_as_ (-CH_3_), δ_as_ (-OCH_3_), 1447 δ_s_ (-OCH_3_), 1416 ν (C-C=C, -Ph1,2,4), 1358 δ_s_ (-CH_3_), 1300 ν (C-C=C, -Ph_ortho_), ν (C-N), secondary amide III, 1256 δ (-Csp^2^-H, -Ph1,2,4), 1157 δ (-Csp^2^-H, -Ph_ortho_), ρ(-CH-)/ν (N-C) in –NH-CH-, 1139 δ (-Csp^2^-H, -Ph1,2,4), 1029 δ (-Csp^2^-H, -Ph_ortho_); HRMS electrospray ionization (ESI) *m*/*z* calcd. for [M+H]^+^ C_18_H_23_O_3_N_2_^+^ = 315.17032, found 315.16956 (mass error ∆m = −2.41 ppm).

#### Synthesis of MA-Loaded AgNPs (1)

Drug-loaded AgNPs were synthesized according to a previously reported method [38]. Then, 1.25 g (0.007 mol) fructose was dissolved in 25 mL of water and refluxed for 2 min; then, 0.63 mL of 0.01 M AgNO_3_ solution was added. Then, MA (**2**) was added at a 2 mg/mL concentration. The solution’s color changed to a light yellow after roughly 5 min of reflux, signifying the formation of (**1**). The final concentration of drug-loaded AgNPs was 2 mg/mL, and this concentration was used for all biological tests.

### 2.2. Antimicrobial Activity Assay

The antimicrobial activity of drug-loaded AgNPs, plain AgNPs, and MA in a concentration of 2 mg/mL was evaluated against Gram-positive bacteria—*Bacillus subtilis* ATCC 6633, *Bacillus cereus* ATCC 11145, *Enterococcus faecalis* ATCC 29212, *Staphylococcus aureus* ATCC 25923, *Listeria monocytogenes* NBIMCC 8632, Gram-negative bacteria—*Salmonella typhimurium* ATCC 13076, *Klebsiella pneumoniae* ATCC 13883, *Escherichia coli* ATCC 25922, *Proteus vulgaris* ATCC 6380, *Pseudomonas aeruginosa* ATCC 9027, two yeasts, *Candida albicans* NBIMCC 74, *Saccharomyces cerevisiae* ATCC 9763, and three fungi, *Aspergillus niger* ATCC 1015, *Penicillium chrysogenum*, and *Fusarium moniliforme* ATCC 3893, using the agar diffusion method [48]. A suspension of each test microorganism (10^8^ cfu/cm^3^) was spread on the surface of preliminarily melted and tempered at 45–48 °C LBG/MEA media and transferred in a quantity of 18 mL in sterile Petri plates (d = 90 mm) (Gosselin, France). Wells of 6 mm diameter were made in the inoculated agar medium. A quantity of 60 μL of the tested substance solution (1 mg/cm^3^ in methanol) was pipetted into the wells. The Petri dishes were then incubated at appropriate temperatures (37 °C for bacteria and *C. albicans* and 30 °C for *S. cerevisiae*) for 24–48 h. The fungi *A. niger*, *A. flavus*, *Penicillium* sp., *Rhizopus* sp., *Mucor* sp., and *F. moniliforme* were grown on Malt Extract Agar at 30 °C for 7 days or until sporulation. After incubation, the inhibition zones around each well were measured, with zones more significant than 7 mm considered inhibition zones. Each test was performed in triplicate, and the results were reported as mean values of the inhibition zone diameters.

Antimicrobial activity was determined by measuring the diameter of the inhibition zones around the wells during the 24 h and 48 h incubation. The tested microorganisms with inhibition zones of 18 mm or more were considered sensitive; those in which the zones were from 12 to 18 mm were considered moderately sensitive, and those in which the inhibition zones were up to 12 mm or completely missing were considered resistant. Two well-known antibiotics, Ampicillin and Nystatin, were used as positive controls.

### 2.3. Inhibition of Albumin Denaturation

The anti-denaturation assay was carried out as described by Milusheva et al. [49,50]. The reaction mixture contained 0.5 mL of a 5% aqueous solution of human albumin (Albunorm 20, Octapharma (IP) SPRL, 1070 Anderlecht, Belgium) and 0.2 mL of the tested samples, drug-loaded AgNPs or MA, in a concentration of 2 mg/mL. The samples were incubated at 37 °C for 15 min. Each tube was filled with 2.5 mL of phosphate-buffered saline (pH 6.3), heated for 30 min to 80 °C, and then cooled for 5 min. The turbidity of the samples was measured spectrophotometrically at 660 nm (Cary 60 UV-Vis, Agilent Technologies, Santa Clara, CA 95051, USA). A mixture of 2.5 mL of buffer and 0.2 mL of DMSO was used for the blank, while the product control contained 0.5 mL of serum albumin and 2.5 mL of buffer.

The percentage inhibition of protein denaturation was calculated according to the following formula:

$$Percentage\;of\;inhibition\;denaturation=\frac{\left(Absorbance\;control-Absorbance\;sample\right)}{Absorbance\;control}\times 100$$

The control represents 100% protein denaturation. Commercially available anti-inflammatory drugs, acetylsalicylic acid and diclofenac, in the same concentration range, were used as positive controls for comparison. Their anti-inflammatory effect was determined as a percentage of inhibition of albumin denaturation, following the same protocol as for MA and the drug-loaded AgNPs.

### 2.4. Ex Vivo Anti-Inflammatory Activity

#### 2.4.1. Immunohistochemical Analysis

After dissection, the smooth muscle strips were incubated with 2mg/mL of the tested compounds for 20 min. Then, each strip was fixed in 10% neutral buffer formalin for 24–48 h. Formalin-fixed, paraffin-embedded tissue sections (5 μm) were deparaffinized after heating at 90 °C for a minimum of 20 min. Immunohistochemical staining was performed with antibodies against IL-1β and nNOS (Elabscience Biotechnology Inc., Houston, TX, USA) using Autostainer Link 48 (Dako, Agilent Technologies, Glostrup, Denmark) using ready-made FLEX antibodies and EnVision FLEX imaging kit.

#### 2.4.2. Histological and Morphometric Analysis

This study was performed on 5 μm sections of the stomach’s circular and longitudinal muscle layers. The sections were stained with IL-1β (E-AB-52153) and 5HT3 (1:300, E-AB-32268) antibodies (Elabscience Biotechnology Inc., Houston, TX, USA). Micrographs were taken with a Leica DM1000 LED and ICC50W camera. The intensity of the reaction was assessed in arbitrary units (0–256 AU) using DP–Soft 3.2 (Olympus, Tokyo, Japan), by analyzing a minimum of 50 measurement points per slice, from five representative slices obtained from a single animal, at 400× magnification.

### 2.5. Ex Vivo Spasmolytic Activity

This study was performed on gastric smooth muscles (SMs) of male Wistar rats weighing 270–290 g. Three or four muscle strips were taken from a rat’s stomach. The number of muscle preparations used for each data point is indicated by n. The isolated SM preparation was placed upright in a thermostated vessel for isolated tissues 15 mL in volume and oxygenated with physiological fluid at a temperature of 37 °C. SM preparations of circular dissection, 12–13 mm in length and 1.0–1.1 mm in width, were used to record isometrically the contractile activity. All transducers used in our experiments were made by the Tissue Organ Bath System (159920 Radnoti, Dublin, Ireland) according to a previously described procedure [39,46,47]. The vitality of the SM tissues was tested by administering 10⁻^6^ M Acetylcholine (ACh) both at the beginning of the experiment and after each application of the substances being tested. 

The initial concentration of drug-loaded AgNPs was 2 mg/mL. Further dilutions were made in distilled deionized water and were applied by aliquots of their concentrated form. The final concentration of the studied compounds did not exceed a 1:100 ratio in the tissue baths.

### 2.6. DFT Calculations

**Figure 2:**
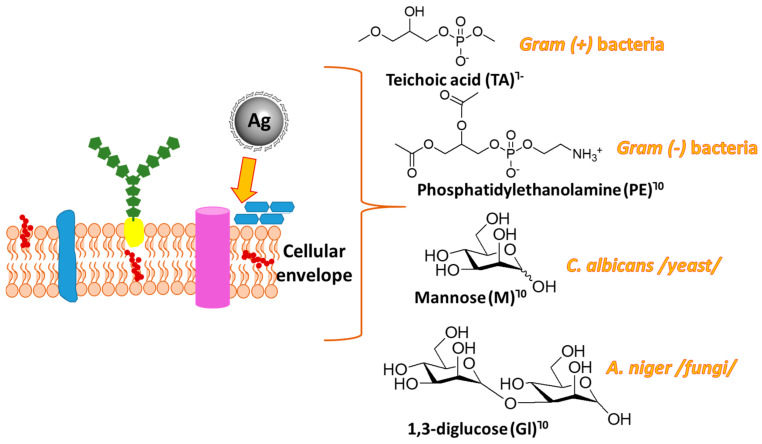
Models used in the current work to study the interaction between silver cations (in the form of Ag(H_2_O)_4_) and the constituents building the outer layer of the microorganism cells [34].

It is known that AgNPs/Ag^+^ exert therapeutic effect by (1) denaturing/inactivating cellular proteins and enzymes, (2) increasing the concentration of reactive oxygen species (ROS), and (3) forming inactive structures when electrostatically interacting with the complementary bases of the DNA double helix. [51]. The Density Functional Theory approach presented in the current study is encoded in the Gaussian 09 Suite of Programs [52] to assess another possible mechanism of silver’s antifungal/antibacterial activity focused on its interaction with the outer envelope of the microorganism cells. AgNPs with smaller particle sizes possess a more significant antimicrobial effect assigned to increased available surface area per unit mass, which allows purely mechanical destruction of the cell wall/membrane, a higher release of Ag^+^, and facilitated interplay with other particles. The expulsed particles in the surrounding media cations are hypothesized to bind the constituents of the external envelope effectively. Hence, we modeled some of the most common constituents in the Gram (+) bacterial wall—teichoic acid (TA); in the Gram (-) outer membrane—phosphatidylethanolamine (PE), known to build about 75% of the membrane lipids in *E. coli*; models of the carbohydrate outer layers in *Candida albicans* known as mannan [53], with reduced structure to mannose; and in *A.* spp.—1,3—β-glucan, calculated as a 1,3-β-glucose dimer [54]. These models are presented in the following Figure 2.

The structures under study, as well as the resulting Ag-containing complexes, were subjected to complete geometry optimization at the M062X/6-311++G(d,p) level of theory for the lighter atoms and SDD pseudopotential for the heavier Ag^+^ found most suitable for the investigated systems in our previous research [55]. The most stable complexes were considered among the different compositions of the Ag-containing complexes. For every structure, the vibrational frequency analysis produced only positive values, indicating a local minimum on the PES (potential energy surface). The corresponding change in the Gibbs energy in the gas phase, ΔG^1^, could, therefore, be calculated according to the main principle of thermodynamics [56] as follows:

ΔG^1^ = ΔE_elect_ + ΔE_T_ + PΔV − TΔS,

where the obtained optimization electronic energies, E_elect_, thermal energies, including zero point energy, E_T_, and entropy, S, were further retrieved and included in the equation. PΔV is a work term that accounts for the change in the number of molecules during a reaction. The overall change in the Gibbs energy in aqueous solution, ΔG^78^, could then be computed as follows:

ΔG^78^ = ΔG^1^ + ΔG^78^_solv_ (products) − ΔG^78^_solv_ (reagents),

where the corresponding solvation energies were calculated at the SMD level of theory available in Gaussian 09 [57], and the experimental value for the water solvation energy was included [58]. Therefore, the growth inhibition effect of the silver ions could be attributed to thermodynamically justified interaction with the constituents of the cellular envelope if the change in the Gibbs energy was negative. In contrast, a positive one suggests an improbable reaction.

### 2.7. Cell Culturing Conditions and IC50 Determination for AgNPs, MA, and AgNPs Loaded with MA

HepG2 cells, derived from human liver carcinoma, were cultured in Dulbecco’s Modified Eagle Medium (DMEM) supplemented with 10% fetal bovine serum (FBS; Sigma-Aldrich, Darmstadt, Germany). The cells were maintained at 37 °C in a humidified incubator with 5% CO_2_. A solution containing 0.05% trypsin and 0.02% EDTA (Sigma-Aldrich, Germany) was used to detach the cells for further experiments. Cytotoxicity evaluations were assessed as previously described [38]. HepG2 cells of AgNPs, MA, and drug-loaded AgNPs were treated in their IC_50_ concentrations for 24 and 72 h [38]. Absorbance was recorded at 450 nm using a Thermo Scientific Multiskan Spectrum ELISA reader (Thermo Scientific, Tokyo, Japan). GraphPad software (GraphPad Prism, version 10.0.3; GraphPad Software, San Diego, CA, USA) determined the half-maximal inhibitory concentration (IC_50_) from dose–response curves.

### 2.8. Fluorescence-Activated Cell Sorting (FACS) Analysis of HepG2 Cells

Flow cytometry evaluated cell cycle progression and morphological alterations in HepG2 cells exposed to AgNPs, AgNPs loaded with MA, and MA in their respective IC_50_ concentrations [38] for 24 and 72 h. Cells were fixed in 76% cold ethanol and stored at −20 °C for 24 h. After fixation, the cells were centrifuged, rinsed with PBS, and incubated with RNase A (100 µg/mL) at 37 °C for 30 min to degrade RNA. For DNA staining, a propidium iodide (PI) solution (50 µg/mL) was applied for 30 min in the dark. Flow cytometry was conducted with an excitation wavelength of 488 nm, and data from 50,000 recorded events per sample were analyzed using FlowJo™ software (Version 10, Becton Dickinson, Franklin Lakes, NJ, USA).

### 2.9. Statistics

All experiments involving HepG2 cells, including cytotoxicity (IC_50_ determination) and cell cycle progression analysis (FACS), were conducted using four standardized groups: untreated cells (Control), cells treated with the mebeverine analogue alone (MA), cells treated with silver nanoparticles alone (AgNPs), and cells treated with MA-loaded silver nanoparticles (AgNPs with MA). These group names have been unified and are used consistently throughout the manuscript.

A one-sample *t*-test and a Wilcoxon test were used for immunohistochemical analysis. Quantitative data were analyzed using the GraphPad Prism software (GraphPad Software 8.0.1 version, Inc., La Jolla, CA, USA). The asterisk indicates significant differences between groups—*** *p* < 0.001.

The in vitro anti-inflammatory experiment was performed in triplicate, and the results were expressed as mean ± SD (standard deviation).

The statistical analysis for spasmolytic and anti-inflammatory activity was conducted using the SPSS 23.0 software (SPSS Inc., Chicago, IL, USA). Data are presented as mean ± SD (standard deviation). The number of tissue preparations used for each experiment is denoted by n. The independent sample *t*-test assessed the statistical significance between two independent groups, with a significance level set at *p* < 0.05.

The obtained data for cell culturing conditions, IC_50_ determination, and FACS analysis were analyzed using GraphPad Prism version 8.0.1 for Windows (San Diego, CA, USA) and FlowJo™ software (Version 10, Becton Dickinson, Franklin Lakes, NJ, USA).

## 3. Results and Discussion

Biofilms are aggregated bacteria that are adhered to surfaces and encased in an extracellular polymeric matrix. Persister cells are protected from the immune system by the local environment within a biofilm [59]. Any surface, such as hospital water distribution systems or medical implants, can develop these bacterial biofilms [60]. The important pathogen *P*. *aeruginosa* is an example of a bacterium that can be very challenging to remove and cause treatment issues, particularly in biofilm. These days, one of the most enduring problems in the world is antibiotic resistance, and numerous potent medications have not been able to control illnesses.

Therefore, the current study aimed to evaluate the antimicrobial and antifungal activity of previously synthesized drug-loaded AgNPs with an MA.

The AgNPs exhibited excellent antimicrobial activity, and this property can be highly beneficial, especially against microorganisms resistant to conventional antimicrobials [61]. The antibacterial activity of AgNPs against various bacteria results in diverse inhibitory zone diameters reported recently [62]. The potential use of the antispasmodic MBH against *Staphylococcus aureus* was recently examined [63,64,65]. The authors demonstrated that MBH was beneficial in lowering the dosage of antibiotics and reducing the need for subsequent antibiotic administration to patients with various problems, including infection, spasm difficulties, and gastrointestinal ulcers.

### 3.1. Antimicrobial Activity

#### 3.1.1. DFT Analysis

**Figure 3:**
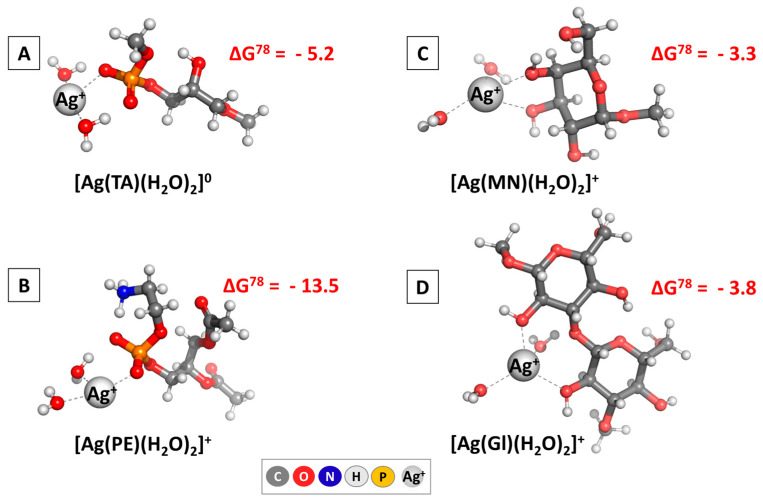
M062X/6-311++G(d,p) optimized structures of the silver-containing complexes of some main constituents building the outer layer of the microorganismic cellular envelope in [**A**] Gram (+) bacteria; [**B**] Gram (−) bacteria; [**C**] *Candida albicans*; [**D**] *Aspergillus* spp. The change in the Gibbs energies in aqueous solution mimicking the intercellular space for yielding these complexes is given in terms of kcal mol^−1^.

The broadly implemented DFT methodology indicated that it is indeed thermodynamically justified for the silver cations to interact with some main constituents, building the outer layer of the microorganisms’ cellular envelope. The results are presented in Figure 3 regarding the change in the Gibbs energy (in kcal mol^−1^).

Irrespective of the intimate composition of the structures under study, the silver cation easily forms complexes, as evidenced by the ΔG^78^ values standing firmly in the negative region. The yielded Gibbs energies vary in the region between −2.6 (for the formation of [Ag(Gl)(H_2_O)_2_]^+^) and −13.5 kcal mol^−1^ (for the [Ag(PE)(H_2_O)_2_]^+^ complex). These data suggest that silver would exert a more pronounced therapeutic effect against Gram (-) bacteria as opposed to Gram (+) bacteria because of the differences in their cell composition, which has indeed been reported in the literature [65]. Moreover, plausible targets in yeast and fungi are expected to be the constituents of their outer shell, modeled here as mannose and 1,3-β-diglucose, with close effects for both microorganisms since the calculated ΔG^78^ do not differ substantially. Note, however, that this interaction path is only one of the many possible mechanisms by which AgNPs/Ag^+^ could prevent microorganisms from growing, and other factors will undoubtedly play an additional role in delineating which microorganism would be most prone to silver-based therapy. Nevertheless, the results presented herewith provide evidence at a molecular level for a thermodynamically justified antifungal and antimicrobial effect of drug-loaded AgNPs.

#### 3.1.2. Experimental Results

The agar diffusion technique was used to examine the antibacterial activity of the drug-loaded AgNPs on five Gram-positive bacteria, five Gram-negative bacteria, two yeasts, and three fungi. The good diffusion method reveals the level of sensitivity in pathogenic microorganisms. Therefore, an organism susceptible to a chemical will not develop near the well where it was placed. The zone of inhibition, also known as the clear zone, is the area that does not grow. The inhibition of the tested substance is proportional to the size of the clear zone.

The mean zones of inhibition in mm produced on the pathogenic microorganisms containing AgNP suspension are presented in Table 1.

Our results illustrated that drug-loaded AgNPs exhibited promising antifungal activity against all tested fungal strains, *Aspergillus niger*, *Penicillium chrysogenum*, and *Fusarium moniliforme*, and yeasts, *Candida albicans*, *Saccharomyces cerevisiae*, and good antimicrobial activity against Gram-positive bacterial strain *Bacillus cereus* and Gram-negative strains, such as *Salmonella typhimurium*, *Escherichia coli*, and *Pseudomonas aeruginosa* (Table 1). This difference in activity can be attributed to the difference in cell membrane permeability between the Gram-positive and Gram-negative bacterial strains, which is significantly affected by complex factors, including the zeta potential on the membrane, the lipophobicity of the cell membrane, the thickness of the membrane and its surrounding layers, and the chemical composition of the antimicrobial drug [66].

*Aspergillus* sp., *Fusarium* sp., *Penicillium* sp., and *Candida* sp. are the most prevalent spoilage fungi. They can produce mycotoxins, which are exceedingly dangerous to people and animals. Grain discoloration, nutritional and chemical alterations, and decreased germination are further signs of spoilage fungi [67,68]. *Aspergillus* sp. is the most common, and up to 22% of air spore samples are Aspergillus spores [69]. Although there are more than 250 *Aspergillus* species, only a few cause issues in humans [70]. The respiratory system is primarily affected by *Aspergillus* infections. Aspergillus spores can germinate in the lungs when high humidity, oxygen, and carbon dioxide are present [71,72,73]. *Aspergillus fumigatus* is the most common species that causes aspergillosis, followed by *A. flavus* and *A. niger*. *A. niger* is among the most common infections that cause mycosis in humans. At least 50% of afflicted individuals can die from systemic invasive infections caused by these molds [74,75]. *A. flavus* produces aflatoxins, which are carcinogenic and mutagenic, and contaminates a range of nuts, fruits, vegetables, and cereals [76]. Long-term exposure to antifungals has been linked to the recent rise of drug-resistant isolates of *Aspergillus* species [77]. In our experiments, drug-loaded AgNPs inhibit *A. niger*, *P. chrysogenum*, and especially *F. moniliforme* growth, showing better potential than plain AgNPs or MA, which is comparable to Nystatin.

*Penicillium* spp. serves as human pathogens, saprophytes, and plant pathogens [78]. *P. chrysogenum* has been exploited by industry for the production of the widely used antibiotic penicillin [79].

*Penicillium chrysogenum*, on the other hand, has been linked to necrotizing pneumonia [80]. Acute respiratory failure with a fatal outcome due to *Penicillium chrysogenum* in a man from Guatemala has also been reported [81]. Our results showed that AgNPs had significant antifungal efficacy against the mycotoxigenic fungus *P. chrysogenum*.

Various serious diseases and recent resistance to the primary antifungals are caused by the yeasts *Candida albicans* and *Candida tropicalis*. The two primary yeasts identified in samples taken from hospitalized patients in the Brazilian state of Ceará are *Candida albicans* and *Candida tropicalis* [82]. *Candida albicans* and *Saccharomyces cerevisiae* showed high sensitivity to drug-loaded AgNPs (Table 1), which can be compared with the activity of Ampicillin, a powerful antibacterial and antifungal drug. Studies highlight this same result with the activity of AgNPs against *Candida* spp. [83].

Recent studies showed that AgNPs in solution may be able to adhere to and saturate fungal hyphae, destroying the fungus cells [84].

The antibacterial qualities of AgNPs are attributed to their special qualities. We obtained smaller spherical drug-loaded AgNPs [38]. The smaller size and wider surface area allow them to interact with bacteria, causing cellular damage and microbial growth inhibition. They can disrupt the cell membrane, interfere with cellular processes, and induce oxidative stress, ultimately resulting in the death of the microorganisms. This can be explained by different cell membrane permeability for both Gram-positive and Gram-negative bacteria, which is greatly influenced by several intricate factors, such as the membrane’s zeta potential, lipophobicity, the thickness of the membrane and its surrounding layers, and the chemical composition of the antimicrobial drug [66].

The experimental results and theoretical calculations allow us to conclude that the drug-loaded AgNPs present promise as excellent drug candidates due to their high antifungal potential and good antimicrobial activity.

### 3.2. In Vitro Anti-Inflammatory Activity

The protein denaturation method has been used to investigate the anti-inflammatory properties of AgNPs. Inflammation results from the process of protein denaturation, which occurs when proteins or nucleic acids lose their quaternary, tertiary, and secondary structures as a result of external stressors or substances like organic solvents, concentrated inorganic salts, strong acid or base, agitation, radiation, or heat [85]. 

AgNPs, on the other hand, have been found in studies to bind to albumin and cause conformational changes that result in denaturation. This is explained by the fact that AgNPs have a high affinity for proteins and can interfere with the hydrophobic and hydrogen bonding interactions that stabilize the protein structure. The denaturation of proteins can also be caused by the oxidative stress that AgNPs produce. Kumari et al. found that AgNPs denature proteins [86].

**Figure 4:**
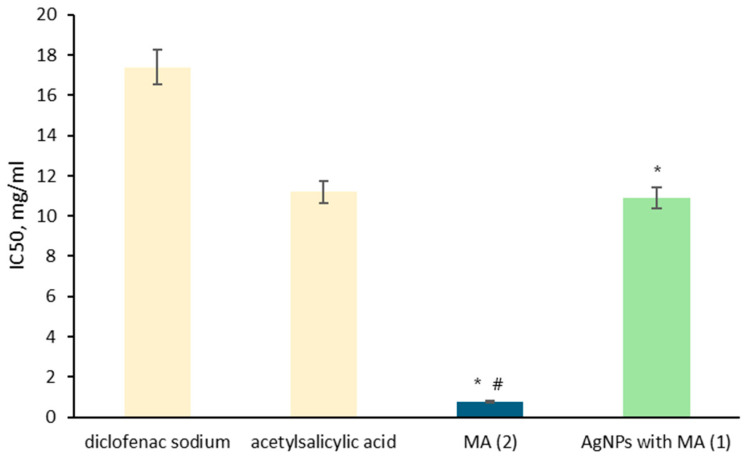
In vitro, the anti-inflammatory activity of (**1**) and (**2**) was assessed to prevent albumin denaturation. (n = 3); # *p* < 0.05 compared to diclofenac; * *p* < 0.05 compared to acetylsalicylic acid.

In our experiments, drug-loaded AgNPs (**1**) have similar protection to acetylsalicylic acid, about two times lower than diclofenac sodium and about 35 times lower than MA (**2**) (Figure 4). The obtained results confirmed the anti-inflammatory activity of MA, which was deposited on the AgNPs’ surface.

There are multiple reasons why AgNPs stabilize cell membranes. First, AgNPs can interact with the membrane’s lipid molecules to create a barrier that lessens membrane disturbance. Second, by scavenging reactive oxygen species (ROS) produced during oxidative stress, they help alleviate the damage that membranes sustain.

### 3.3. Cell Culturing Conditions and IC₅_0_ Determination for AgNPs, MA, and AgNPs Loaded with MA

Given the potential of AgNPs as antimicrobial and anti-inflammatory agents, understanding their cytotoxic effects is crucial for evaluating their therapeutic safety. To address this, the current study examined the impact of AgNPs, MA, and their nano-formulated combination on HepG2 cells, a commonly used in vitro model for cellular toxicity. The IC_50_ values at 24 h indicated that AgNPs were the most cytotoxic (6.35 µg/mL), followed by AgNPs with MA (8.88 µg/mL), while MA alone showed significantly lower toxicity (24.69 µg/mL). After 72 h, IC_50_ values increased for all treatments, suggesting cellular adaptation. AgNPs remained very toxic (17.79 µg/mL), while AgNPs with MA exhibited even more substantial toxicity with 13.93 µg/mL and MA—the lowest toxicity among tested samples with 29.39 µg/mL (Table 2). MA is less cytotoxic than both NPs because it lacks the ROS-mediated and nanoparticle-induced mechanisms that drive toxicity in AgNPs and AgNPs with MA. Its effect on HepG2 cells is more functional and regulatory than disruptive or damaging.

Our findings suggest that MA incorporation in AgNPs modulates their effect, providing valuable data for their potential therapeutic applications.

Several studies have reported varying IC_50_ values for AgNPs on HepG2 cells, which can be attributed to differences in nanoparticle synthesis methods, particle sizes, and surface chemistries. For example, one study reported an IC_50_ of 2.764 µg/mL for AgNPs after 24 h of exposure, indicating higher toxicity than observed in our study [87]. Conversely, AgNPs synthesized using plant extracts demonstrated IC_50_ values ranging from 10.04 to 70.97 µg/mL, depending on the plant source used, highlighting the critical role of nanoparticle characteristics in influencing cytotoxic profiles [88].

Regarding the mebeverine analogue, the existing literature contains limited data on its cytotoxicity, particularly in terms of IC_50_ values. A study by Milusheva et al. [46] demonstrated that newly synthesized mebeverine precursors exhibited no cytotoxicity against human leukemic cell lines (LAMA-84, K-562), although quantitative IC_50_ values were not reported. Consequently, the current study provides novel and valuable quantitative insight into the cytotoxic profile of the mebeverine analogue and its nano-formulated form, emphasizing the need for further exploration of its biomedical potential.

### 3.4. Fluorescence-Activated Cell Sorting (FACS) Analysis of HepG2 Cell Morphology

**Figure 5:**
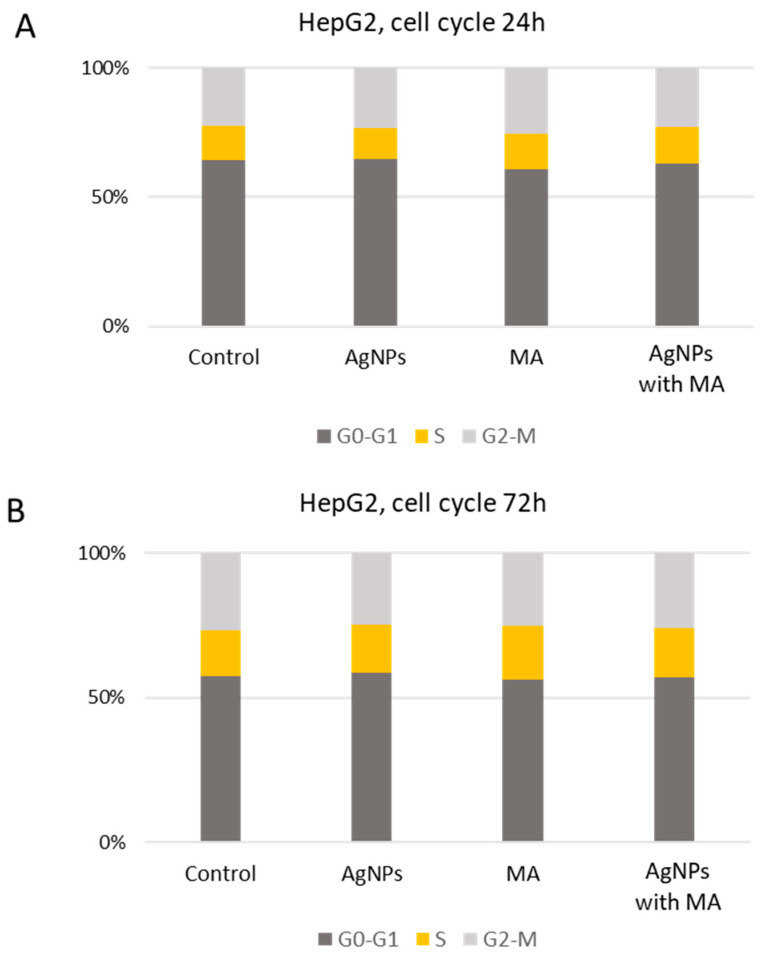
Cell cycle distribution of HepG2 cells treated with AgNPs, MA, and AgNPs with MA for 24 h (**A**) and 72 h (**B**). The percentage of cells in G_0_–G_1_ (dark grey), S (light grey), and G_2_–M (yellow) phases was determined via flow cytometry and subsequent data evaluation using FlowJo™ software (Version 10, Becton Dickinson, USA). The treatments were applied at concentrations corresponding to the IC50 values determined for each compound and time point: MA—24.69 µg/mL (24 h), 29.39 µg/mL (72 h); AgNPs—6.35 µg/mL (24 h), 17.79 µg/mL (72 h); AgNPs with MA—8.88 µg/mL (24 h), 13.93 µg/mL (72 h).

The presented figure (Figure 5) illustrates the impact of AgNPs, MA, and their nano-formulated combination (AgNPs with MA) on HepG2 cell cycle progression after 24 h (A) and 72 h (B) of treatment, compared to control conditions. Flow cytometry analysis reveals the percentage of cells in each cell cycle phase (G_0_–G_1_, S, and G_2_–M), providing insights into potential therapeutic and adverse effects of cell proliferation dynamics and mitotic regulation. Given the antimicrobial and anti-inflammatory properties of AgNPs, understanding their influence on hepatic and intestinal cells is crucial for evaluating their safety and efficacy.

At 24 h, control cells displayed a typical cycle distribution (65.2% in G_0_–G_1_, 13.4% in S, and 22.7% in G_2_–M). AgNP treatment resulted in a reduction in G_0_–G_1_ cells (61.5%) and a slight decrease in S-phase cells (11.6%), suggesting suppressed DNA synthesis, while the G_2_–M phase (22.2%) remained near control levels. In contrast, MA treatment increased G_2_–M cells (28.3%) and S-phase cells (14.7%), implying mitotic progression delays. The AgNPs with MA induced the highest G_0_–G_1_ accumulation (68.8%), along with an increase in S-phase (15.6%) and a moderate reduction in G_2_–M (25.0%), suggesting a cell cycle blockade at S-phase.

At 72 h, the control group maintained a similar profile (63.4% G_0_–G_1_, 17.6% S, and 29.5% G_2_–M). AgNPs further increased G_0_–G_1_ accumulation (68.5%), with a moderate rise in S-phase (19.6%), indicating a persistent inhibitory effect on cell cycle progression. MA treatment led to a decrease in G_0_–G_1_ (60.9%) and an increase in both S-phase (20.2%) and G_2_–M (27.2%), highlighting continued mitotic interference. The most pronounced effect was observed with MA-loaded AgNPs, which led to the highest G_2_–M accumulation (31.4%) and sustained S-phase increase (20.3%), suggesting a prolonged mitotic arrest.

These results indicate that AgNPs predominantly induce G_0_–G_1_ arrest, limiting proliferation, while MA promotes S-phase entry and G_2_–M accumulation, affecting mitotic activity. The MA-loaded AgNP combination exhibits a dual effect, enhancing S-phase and G_2_–M accumulation, suggesting an interplay between cell cycle arrest and mitotic disruption.

Compared to previous studies, our findings align with the well-documented cytotoxicity of AgNPs in liver-derived cell lines, where cell cycle arrest is reported as one of the mechanisms of growth inhibition [87]. However, the observed dual-phase accumulation in the S and G_2_–M phases with AgNPs with MA appears to be a novel or less frequently reported effect, suggesting that the combination with MA enhances cell cycle interference beyond what is typically seen with AgNPs or MA alone. This dual-phase disruption may offer a distinct therapeutic advantage, particularly in cancer treatment, where targeting multiple points of cell cycle progression can increase efficacy and reduce resistance.

### 3.5. Immunohistochemistry, Ex Vivo Anti-Inflammatory Effect

The drug-loaded AgNPs were synthesized as drug candidates, targeting gut microbiota and inflammation. Therefore, we also attempted to evaluate the anti-inflammatory effect of drug-loaded AgNPs (**1**), compared to MA (**2**), by measuring the intensity and density of IL-1β and 5HT3 expression.

The release of cytokines in response to various disease states, such as IBD, increases the levels of pro-inflammatory cytokines such as IL-1, IL-6, and IL-17, significantly enhancing intestinal inflammation [89].

Using drug-loaded NPs to deliver drugs and accumulate them at the site of inflammation through their uptake by immune cells is one of the goals of selective delivery [90].

**Figure 6:**
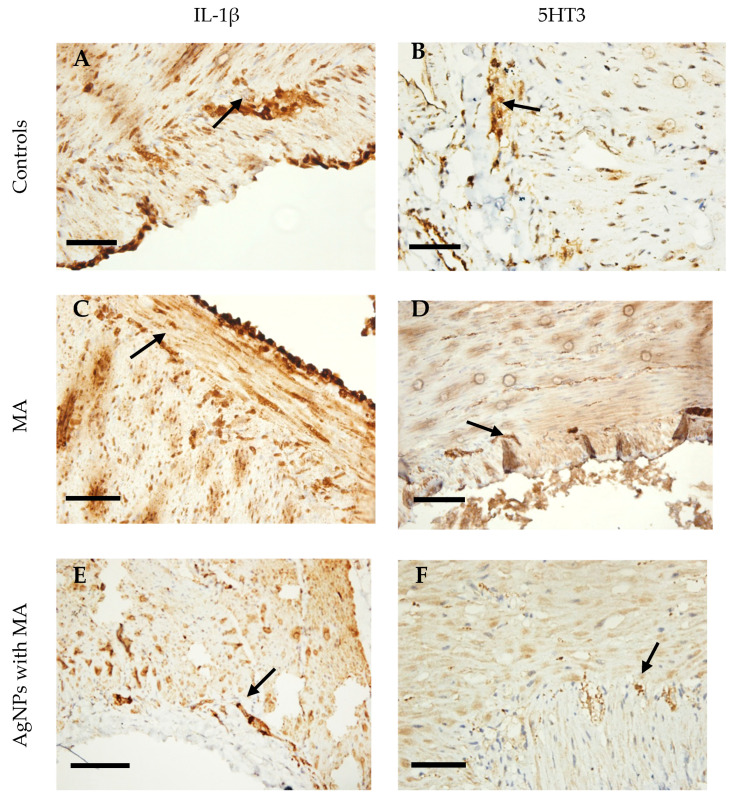
Representative micrographs of the rat corpus stomach. (**A**) Control stained for IL-1β, ×400; (**B**) Control stained for 5HT3, 400×; (**C**) samples incubated with MA show a very high density of expression for IL-1β in the myenteric plexus (black arrow), 400×; (**D**) MA showed a moderate level of 5HT3 expression (black arrow), 400×; (**E**) AgNPs with MA caused weak staining of cells for IL-1β (black arrow), 400×; (**F**) drug-loaded AgNPs very weakly labeled cells for 5HT3 (black arrow), 400×; scale bar = 50 μm.

In comparison to the controls (Figure 6A), MA (173.6 ± 3.2) showed a higher intensity and density of expression of IL-1β (Figure 6C) than AgNP with MA (123.7 ± 2.7 AU) (Figure 6E).

AgNPs with MA (130±6.3 AU), on the other hand, significantly reduced the staining intensity of the cells for 5HT3 (Figure 6F), whereas MA (152 ± 3.1 AU) promoted lowering the expression of 5HT3 in the myenteric plexus neurons below the controls (Figure 6D).

The results of the comparative study of 5-HT3 receptor expression are consistent with earlier studies showing that 5-HT3 receptor antagonists (such as ramosetron and ondansetron) alleviate intestinal damage [91,92]. According to Maehara et al., 5-HT3 receptor antagonists effectively reduced the severity of postoperative ileus and colitis [93].

Thus, drug-loaded AgNPs are crucial for evaluating the MA’s anti-inflammatory properties and its effects on serotonin receptors, especially the 5-HT3 channel receptor, regarding neurotoxicity. This could be useful for drug delivery and identifying monoamine neurotransmitters [94].

The results of the immunohistochemical analysis indicated that AgNPs with MA preserve the activity of MA in its robust suppression of 5HT3 expression, which helps mitigate intestinal damage and significantly diminishes IL-1β expression and, consequently, inflammation.

### 3.6. Evaluation of Ex Vivo Spasmolytic Effect

The reactivity of SMs is determined by their structural characteristics and the availability of Ca^2^⁺ ions. [95]. Their function is influenced by various factors that either activate or inhibit specific signaling pathways, resulting in either contraction or relaxation of the SM. The key regulatory element in both scenarios is the cell’s free ionized Ca^2^⁺ ions concentration. It is well established that Ca^2^⁺ homeostasis can be influenced by substances that act via G proteins, which activate phospholipase C and produce secondary messengers such as IP_3_ and DAG [96]. As a result, IP_3_ triggers the release of Ca^2^⁺ from intracellular stores by binding to IP_3_ receptors located on the endoplasmic reticulum. Another mechanism for Ca^2^⁺ release involves the activation of ryanodine receptors on the endoplasmic reticulum [97]. One of the most well-understood pathways for Ca^2^⁺ entry is through ligand-gated Ca^2+^ channels, which various ligands activate [98]. Contraction of SM generally involves a combination of Ca^2^⁺ release and influx, which are triggered by neurotransmitters (such as ACh, 5-HT, Carbachol, etc.) or hormones [99].

MA (**2**) was synthesized as a spasmolytic. Therefore, our next goal was to determine whether the spasmolytic activity of MA deposited on the drug-loaded AgNPs was changed. To elucidate the specific effects of the drug-loaded AgNPs compared to MA (**2**) on SM contractility, we examined its interactions with various cholinergic inhibitors (hexamethonium, decamethonium), selective cholinergic antagonists (pirenzepine, gallamine, 4-DAMP), Ca^2^⁺ blockers (atropine and ipratropium), and key neurotransmitters (ACh, 5-HT) [100,101].

In the present study, the impact on the main mechanokinetic parameters of spontaneous SM contractile activity was followed using isometric measurements and subsequent analysis. Thus, the tonus, amplitude, and frequency changes were evaluated under the influence of (**2**) and AgNPs used as a drug delivery system (**1**). The applied volume of the tested substance was between 2.5 and 20 μL. The conventional approach using in vitro and ex vivo models involved examining the dose–response relationship to determine the biological effect. In this way, the effect of the tested compounds was characterized.

In the current study, an isolated SM model from the rat stomach was used to evaluate the biological effects of AgNPs as modulators of muscle activity. Initially, the viability of the isolated SM tissues was assessed based on their reaction to ACh. After evaluating cumulative dose-dependent effects, we observed significant differences in the mechanical responses of SM preparations treated with (**2**) and drug-loaded AgNPs (**1**) (Table 3).

After applying (**2**), a pronounced relaxation effect was observed, leading to a substantial reduction in muscle tonus and amplitude. In contrast, (**1**) exhibited a weaker effect, with changes in the baseline state of SM limited to approximately 20%. Notably, neither compound caused significant alterations in the frequency of spontaneous alternating contraction and relaxation peaks.

The observed differences suggest that the effects on the cellular processes regulating spontaneous SM responses occur through distinct mechanisms. It is well established that spasmolytic activity is not confined to a single pathway but exhibits polyvalent effects involving at least three primary mechanisms. These include a direct myotropic action associated with Ca^2^⁺ ion exchange, a competitive antimuscarinic activity, and a local anesthetic effect that inhibits norepinephrine reuptake. On the other hand, individual application of AgNPs does not result in a significant change in the tonus of isolated SM from the trachea. However, when AgNPs are administered simultaneously with ACh, a slight increase in ACh-induced contractility is observed, after which the muscle tonus fails to return to its baseline level. According to previous studies, this effect is attributed to increased nitric oxide production, which may play a key role in muscle activity regulation [102].

**Figure 7:**
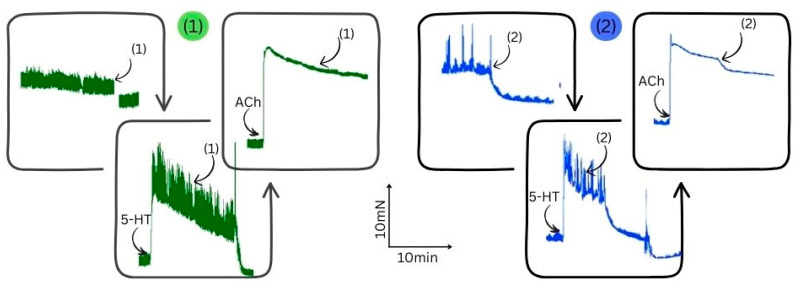
Representative isometric tracings illustrating the contractile behavior of gastric SM in response to ACh (10⁻^6^ M) and 5-HT (10⁻^6^ M), and the modulatory effects of compounds (**1**) and (**2**) in their background.

To further investigate the mechanism of action of (1) on the cellular processes of SM, we decided to apply two key neurotransmitters—ACh and 5-HT—in the tissue baths before and after AgNPs’ application (Figure 7). Thus, we observed their mutual influence. Following cholinergic or serotonergic stimulation, the muscle preparations exhibited a sharp increase in tonus (phasic contraction) associated with elevated intracellular Ca^2^⁺ ion levels. The subsequent sustained tonic contraction was driven by Ca^2^⁺ influx, which triggered Ca^2^⁺ oscillations. Interestingly, this contractile activity pathway does not appear to be significantly affected by drug-loaded AgNPs. Preincubation of tissues with (**1**) and (**2**) did not produce substantial changes in the responses to either neurotransmitter. However, (**2**) demonstrated the ability to reduce the strength of the cholinergic response by 32%, likely due to the absence of NPs in its structure, in contrast to (**1**) [103,104].

**Figure 8:**
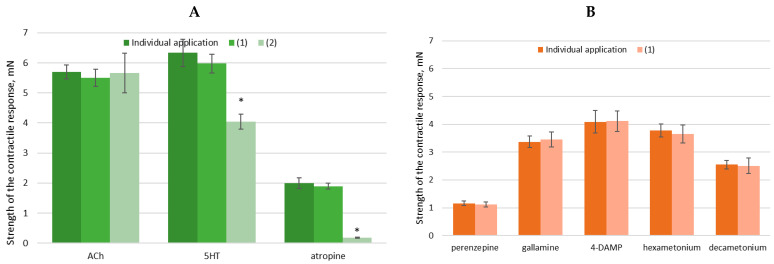
Changes in the strength of the contractile response of SM under the influence of compounds (**1**) and (**2**) and key neurotransmitters. (**A**) Effects in the presence of ACh, 5-HT, and atropine (a non-selective muscarinic receptor blocker). (**B**) Modulation of contractile responses by selective and non-selective antagonists of muscarinic (mAChRs) and nicotinic (nAChRs) ACh receptors. Results are presented as mean ± SEM (n = 6); * *p* < 0.05 indicates statistical significance.

Due to the potential differences in the mechanisms of drug-loaded AgNPs, which can lead to either contractile or relaxing effects on SM, we further investigated changes in the strength of the contractile response upon the application of NPs (at a volume of 20 μL), in the presence of blockers and activators of spontaneous contractility (Figure 8A). The ex vivo tissue bath model allows for the independent or combined exogenous application of these substances, enabling the monitoring and analysis of the changes in the electrical, pharmacological, and electropharmacological responses of treated tissue segments.

A key additional finding is that blocking mAChRs with the non-selective antagonist atropine (10⁻^6^ M, n = 6) does not significantly alter the effects of AgNPs but leads to a 90% reduction in the tonic relaxing effect of (**2**). This highlights the importance of the distinct molecular pathways through which the two compounds exert their effects on SM.

To gain a deeper understanding of the mechanism of action of substances encapsulated in AgNPs, additional studies were conducted using both selective and non-selective antagonists of mAChRs and nAChRs. Upon inhibition of these receptors through specific blockers—pirenzepine (10⁻^5^ M, selective for M1 mAChRs), gallamine (10⁻^5^ M, selective for M2 mAChRs), 4-DAMP (3 × 10⁻^7^ M, selective for M3 mAChRs), hexamethonium (10⁻^5^ M, nicotinic AChR blocker), and decamethonium (10⁻^5^ M, nicotinic AChR blocker)—no significant changes in the responses to (**2**) were induced (Figure 8B). When AgNPs were applied in the presence of selectively blocked cholinergic receptors, their effect exhibited similar intensity and characteristics in their activity.

Applying drug-loaded AgNPs with selectively blocked cholinergic receptors—both mAChRs and nAChRs—reveals a distinct response profile, indicating that the NPs influence receptor-level interactions. Notably, while atropine markedly suppresses the relaxing effect of MA, it does not significantly alter the activity of drug-loaded AgNPs, further supporting the hypothesis that their biological activity arises from a separate or modified mechanism of action.

Finally, we can conclude that the drug-loaded AgNPs preserved the spasmolytic activity of MA, activating both mAChRs and nAChRs Acetylcholine receptors. Taken together, the results suggest that the deposition of MA into AgNPs alters its pharmacological profile, possibly through modifications in receptor targeting or intracellular signaling.

## 4. Conclusions

Our results provide valuable insights into the biological activity of the drug-loaded AgNPs. The antimicrobial activity of drug-loaded AgNPs was investigated against Gram-positive and Gram-negative bacteria, yeasts, and fungi. The AgNPs showed a high antimicrobial capability. AgNPs were considered to have promising bactericidal and fungicidal properties. Future in vivo and preclinical experiments will contribute to the establishment of drug-loaded AgNPs as potential antimicrobial and antifungal agents. Further work is necessary to elucidate in detail the mechanism of action of AgNPs at the cellular and molecular levels.

To determine whether drug-loaded AgNPs can disrupt the intestinal balance, their anti-inflammatory, spasmolytic activity, and hepatic cell morphology and proliferation were also assessed. The drug-loaded AgNPs strongly suppress 5HT3 expression and reduce the intensity of IL-1β expression but have a lower effect on albumin protection than diclofenac.

The drug-loaded AgNPs exhibit a mild modulatory effect on SM contractility, characterized by a modest tonic relaxation and a reduction in spontaneous contractions by approximately 20%. In contrast, MA exerts a significantly more relaxing effect, decreasing both the tonus and amplitude of contraction and reducing cholinergic-induced contractile responses by 32%. The biological activity of the drug-loaded AgNPs may thus be attributed to a complex interplay involving mAChR and nAChR activation, highlighting their potential as multifunctional modulators of SM tonus but still maintaining the spasmolytic activity of MA.

Based on the results, drug-loaded AgNPs might be a promising antimicrobial agent, which maintains the MA’s potential as a spasmolytic and anti-inflammatory. Future in vivo and preclinical experiments will contribute to establishing the in vivo properties of drug-loaded AgNPs.

Regarding their cytotoxic potential, the incorporation of MA into AgNPs appears to modulate both the cytotoxicity and genotoxicity of the nanoparticles. AgNPs-MA represent a balanced or synergistically modulated system, where MA weakens the cytotoxicity of AgNPs, and the cell cycle effects are intensified, likely due to complementary mechanisms of both agents. This intermediate behaviour suggests that nano-formulation can fine-tune the biological activity of AgNPs, which is particularly promising in targeted cancer therapy, where you want controlled toxicity and effective cell cycle disruption.

## Figures and Tables

**Table 1:** Inhibition zone diameter (mm) of the drug-loaded AgNPs (**1**) compared to the MA (**2**).

	Drug-Loaded AgNPs (1)	AgNPs	MA (2)	N *	A *
*Bacillus subtilis*, ATCC 6633	10	ND	ND	n.a.	16
*Bacillus cereus* NCTC 11145	12	ND	ND	n.a.	20
*Staphylococcus aureus*, ATCC 25923	8	ND	ND	n.a.	35
*Listeria monocytogenes*, NBIMCC 8632	11	ND	ND	n.a.	40
*Enterococcus faecalis*, ATCC 29212	9	ND	10	n.a.	38
*Salmonella typhimurium*, NBIMCC 1672	12	ND	ND	n.a.	40
*Klebsiella pneumoniae*, ATCC 13883	11	8	10	n.a.	25
*Escherichia coli*, ATCC 25922	12	9	ND	n.a.	16
*Proteus vulgaris*, ATCC 6380	11	11	10	n.a.	30
*Pseudomonas aeruginosa*, ATCC 9027	12	8	ND	n.a.	16
*Candida albicans*, NBIMCC 74	15	ND	12	21	n.a.
*Saccharomyces cerevisiae*, ATCC 9763	15	ND	ND	18	n.a.
*Aspergillus niger*, ATCC 1015	15	14	ND	18	n.a.
*Penicillium chrysogenum*	16	11	ND	n.a.	n.a.
*Fusarium moniliforme*, ATCC 38932	14	ND	ND	15	n.a.

* Controls: N—Nystatin; A—Ampicillin. ND means no activity was detected, n.a.—not applicable.

**Table 2:** IC_50_ values of the tested compounds **1**–**5** against HepG2 cells at the 24th h and 72nd h.

Samples	IC50 at 24th h [µg/mL]	IC50 at 72nd h [µg/mL]
MA	24.69	29.39
AgNPs	6.35	17.79
AgNPs with MA	8.88	13.93

**Table 3:** Quantitative evaluation of spontaneous contractile activity in isolated SM tissues: effects of compounds (**1**) and (**2**) on basal muscle tonus (mN), contractile amplitude (mN), and frequency (n/min) across varying concentrations and volumes.

Concentration, M	Drug-Loaded AgNPs (1)			Volume, µL	MA (2)		
	Tonus, mN	Amplitude, mN	Frequency, mN		Tonus, mN	Amplitude, mN	Frequency, mN
**10^−7^**	2.2	4	4.7	**2.5**	2	5	4
**5 × 10^−6^**	2.2	4	4.8	**5**	2	5	3.8
**2.5 × 10^−6^**	2	3.8	4.8	**7.5**	1.8	4.3	3.8
**10^−6^**	2	3.7	4.6	**10**	1.5	4	3.9
**2.5 × 10^−5^**	2	3.6	4.7	**12.5**	1.3	3.2	3.7
**5 × 10^−5^**	1.8	3.7	4.7	**15**	1	3	3.7
**10^−5^**	1.8	3.7	4.8	**17.5**	0.5	2	3.6
**5 × 10^−4^**	1.7	3.6	4.5	**20**	0.2	1	3.6

## Data Availability

The data presented in this study are available on request from the corresponding author.

## References

[1] Bengmark S. (1998). Ecological Control of the Gastrointestinal Tract. The role of probiotic flora. Gut.

[2] Li X.V., Leonardi I., Iliev I.D. (2019). Gut Mycobiota in Immunity and Inflammatory Disease. Immunity.

[3] Rooks M.G., Garrett W.S. (2016). Gut Microbiota, Metabolites and Host Immunity. Nat. Rev. Immunol..

[4] Bajinka O., Darboe A., Tan Y., Abdelhalim K.A., Cham L.B. (2020). Gut Microbiota and the Human Gut Physiological Changes. Ann. Microbiol..

[5] Lee J.-Y., Tsolis R.M., Bäumler A.J. (2022). The Microbiome and Gut Homeostasis. Science.

[6] Siezen R.J., Kleerebezem M. (2011). The Human Gut Microbiome: Are We Our Enterotypes?. Microb. Biotechnol..

[7] Markey L., Shaban L., Green E.R., Lemon K.P., Mecsas J., Kumamoto C.A. (2018). Pre-Colonization with the Commensal Fungus *Candida albicans* Reduces Murine Susceptibility to *Clostridium difficile* Infection. Gut Microbes.

[8] Zhang F., Aschenbrenner D., Yoo J.Y., Zuo T. (2022). The Gut Mycobiome in Health, Disease, and Clinical Applications in Association with the Gut Bacterial Microbiome Assembly. Lancet Microbe.

[9] Frey-Klett P., Burlinson P., Deveau A., Barret M., Tarkka M., Sarniguet A. (2011). Bacterial-Fungal Interactions: Hyphens between Agricultural, Clinical, Environmental, and Food Microbiologists. Microbiol. Mol. Biol. Rev..

[10] Rao C., Coyte K.Z., Bainter W., Geha R.S., Martin C.R., Rakoff-Nahoum S. (2021). Multi-Kingdom Ecological Drivers of Microbiota Assembly in Preterm Infants. Nature.

[11] Zuo T., Wong S.H., Cheung C.P., Lam K., Lui R., Cheung K., Zhang F., Tang W., Ching J.Y.L., Wu J.C.Y. (2018). Gut Fungal Dysbiosis Correlates with Reduced Efficacy of Fecal Microbiota Transplantation in *Clostridium difficile* Infection. Nat. Commun..

[12] Chang J.T. (2020). Pathophysiology of Inflammatory Bowel Diseases. N. Engl. J. Med..

[13] Milani C., Duranti S., Bottacini F., Casey E., Turroni F., Mahony J., Belzer C., Delgado Palacio S., Arboleya Montes S., Mancabelli L. (2017). The First Microbial Colonizers of the Human Gut: Composition, Activities, and Health Implications of the Infant Gut Microbiota. Microbiol. Mol. Biol. Rev..

[14] Zhao J., Zhang X., Liu H., Brown M.A., Qiao S. (2018). Dietary Protein and Gut Microbiota Composition and Function. Curr. Protein Pept. Sci..

[15] Li J., Chen D., Yu B., He J., Huang Z., Mao X., Zheng P., Yu J., Luo J., Tian G. (2020). The Fungal Community and Its Interaction with the Concentration of Short-Chain Fatty Acids in the Faeces of Chenghua, Yorkshire and Tibetan Pigs. Microb. Biotechnol..

[16] Lam S., Zuo T., Ho M., Chan F.K.L., Chan P.K.S., Ng S.C. (2019). Review Article: Fungal Alterations in Inflammatory Bowel Diseases. Aliment. Pharmacol. Ther..

[17] Bongomin F., Gago S., Oladele R., Denning D. (2017). Global and Multi-National Prevalence of Fungal Diseases—Estimate Precision. J. Fungi.

[18] Robbins N., Caplan T., Cowen L.E. (2017). Molecular Evolution of Antifungal Drug Resistance. Ann. Rev. Microbiol..

[19] Thambugala K.M., Daranagama D.A., Tennakoon D.S., Jayatunga D.P.W., Hongsanan S., Xie N. (2024). Humans vs. Fungi: An Overview of Fungal Pathogens against Humans. Pathogens.

[20] Roy M., Karhana S., Shamsuzzaman M., Khan M.A. (2023). Recent Drug Development and Treatments for Fungal Infections. Braz. J. Microbiol..

[21] Rabaan A.A., Sulaiman T., Al-Ahmed S.H., Buhaliqah Z.A., Buhaliqah A.A., AlYuosof B., Alfaresi M., Al Fares M.A., Alwarthan S., Alkathlan M.S. (2023). Potential Strategies to Control the Risk of Antifungal Resistance in Humans: A Comprehensive Review. Antibiotics.

[22] Roemer T., Krysan D.J. (2014). Antifungal Drug Development: Challenges, Unmet Clinical Needs, and New Approaches. Cold Spring Harb. Perspect. Med..

[23] Lee Y., Robbins N., Cowen L.E. (2023). Molecular Mechanisms Governing Antifungal Drug Resistance. NPJ Antimicrob. Resist..

[24] Slavin Y.N., Bach H. (2022). Mechanisms of Antifungal Properties of Metal Nanoparticles. Nanomaterials.

[25] Huang T., Li X., Maier M., O’Brien-Simpson N.M., Heath D.E., O’Connor A.J. (2023). Using Inorganic Nanoparticles to Fight Fungal Infections in the Antimicrobial Resistant Era. Acta Biomater..

[26] Wahab S., Salman A., Khan Z., Khan S., Krishnaraj C., Yun S.I. (2023). Metallic Nanoparticles: A Promising Arsenal against Antimicrobial Resistance-Unraveling Mechanisms and Enhancing Medication Efficacy. Int. J. Mol. Sci..

[27] Madkhali O.A. (2023). A Comprehensive Review on Potential Applications of Metallic Nanoparticles as Antifungal Therapies to Combat Human Fungal Diseases. Saudi Pharm. J..

[28] Devaraji M., Thanikachalam P.V., Elumalai K. (2024). The Potential of Copper Oxide Nanoparticles in Nanomedicine: A Comprehensive Review. Biotechnol. Notes.

[29] Sandhu Z.A., Raza M.A., Alqurashi A., Sajid S., Ashraf S., Imtiaz K., Aman F., Alessa A.H., Shamsi M.B., Latif M. (2024). Advances in the Optimization of Fe Nanoparticles: Unlocking Antifungal Properties for Biomedical Applications. Pharmaceutics.

[30] Seong M., Lee D.G. (2018). Reactive Oxygen Species-Independent Apoptotic Pathway by Gold Nanoparticles in *Candida albicans*. Microbiol. Res..

[31] Parada J., Tortella G., Seabra A.B., Fincheira P., Rubilar O. (2024). Potential Antifungal Effect of Copper Oxide Nanoparticles Combined with Fungicides against *Botrytis cinerea* and *Fusarium oxysporum*. Antibiotics.

[32] Mosquera-Sánchez L.P., Arciniegas-Grijalba P.A., Patiño-Portela M.C., Guerra–Sierra B.E., Muñoz-Florez J.E., Rodríguez-Páez J.E. (2020). Antifungal Effect of Zinc Oxide Nanoparticles (ZnO-NPs) on *Colletotrichum* sp., Causal Agent of Anthracnose in Coffee Crops. Biocatal. Agric. Biotechnol..

[33] Subba B., Rai G.B., Bhandary R., Parajuli P., Thapa N., Kandel D.R., Mulmi S., Shrestha S., Malla S. (2024). Antifungal Activity of Zinc Oxide Nanoparticles (ZnO NPs) on Fusarium Equiseti Phytopathogen Isolated from Tomato Plant in Nepal. Heliyon.

[34] Achilonu C.C., Kumar P., Swart H.C., Roos W.D., Marais G.J. (2024). Zinc Oxide:Gold Nanoparticles (ZnO:Au NPs) Exhibited Antifungal Efficacy Against *Aspergillus niger* and *Aspergillus candidus*. BioNanoScience.

[35] Aftab Z.-e.-H., Mirza F.S., Anjum T., Rizwana H., Akram W., Aftab M., Ali M.D., Li G. (2025). Antifungal Potential of Biogenic Zinc Oxide Nanoparticles for Controlling Cercospora Leaf Spot in Mung Bean. Nanomaterials.

[36] Zhao G., Stevens S.E. (1998). Multiple Parameters for the Comprehensive Evaluation of the Susceptibility of *Escherichia coli* to the Silver Ion. Biometals.

[37] Sütterlin S., Tano E., Bergsten A., Tallberg A., Melhus H. (2012). Effects of Silver-Based Wound Dressings on the Bacterial Flora in Chronic Leg Ulcers and Its Susceptibility in Vitro to Silver. Acta Derm. Venereol..

[38] Stoyanova M., Milusheva M., Georgieva M., Ivanov P., Miloshev G., Krasteva N., Hristova-Panusheva K., Feizi-Dehnayebi M., Ghodsi M.Z., Stojnova K. (2025). Synthesis, Cytotoxic and Genotoxic Evaluation of Drug-Loaded Silver Nanoparticles with Mebeverine and Its Analog. Pharmaceuticals.

[39] Stoyanova M., Milusheva M., Gledacheva V., Stefanova I., Todorova M., Kircheva N., Angelova S., Pencheva M., Stojnova K., Tsoneva S. (2024). Spasmolytic Activity and Anti-Inflammatory Effect of Novel Mebeverine Derivatives. Biomedicines.

[40] Kurul F., Turkmen H., Cetin A.E., Topkaya S.N. (2025). Nanomedicine: How Nanomaterials Are Transforming Drug Delivery, Bio-Imaging, and Diagnosis. Next Nanotechnol..

[41] Meher A., Tandi A., Moharana S., Chakroborty S., Mohapatra S.S., Mondal A., Dey S., Chandra P. (2024). Silver Nanoparticle for Biomedical Applications: A Review. Hybrid. Adv..

[42] Dhaka A., Mali S.C., Sharma S., Trivedi R. (2023). A Review on Biological Synthesis of Silver Nanoparticles and Their Potential Applications. Results Chem..

[43] Ferdous Z., Nemmar A. (2020). Health Impact of Silver Nanoparticles: A Review of the Biodistribution and Toxicity Following Various Routes of Exposure. Int. J. Mol. Sci..

[44] Fahim M., Shahzaib A., Nishat N., Jahan A., Bhat T.A., Inam A. (2024). Green Synthesis of Silver Nanoparticles: A Comprehensive Review of Methods, Influencing Factors, and Applications. JCIS Open.

[45] Sati A., Ranade T.N., Mali S.N., Yasin H.K.A., Pratap A. (2025). Silver Nanoparticles (AgNPs): Comprehensive Insights into Bio/Synthesis, Key Influencing Factors, Multifaceted Applications, and Toxicity—A 2024 Update. ACS Omega.

[46] Milusheva M., Gledacheva V., Stefanova I., Pencheva M., Mihaylova R., Tumbarski Y., Nedialkov P., Cherneva E., Todorova M., Nikolova S. (2023). In Silico, in Vitro, and Ex Vivo Biological Activity of Some Novel Mebeverine Precursors. Biomedicines.

[47] Milusheva M., Gledacheva V., Batmazyan M., Nikolova S., Stefanova I., Dimitrova D., Saracheva K., Tomov D., Chaova-Gizdakova V. (2022). Ex Vivo and in Vivo Study of Some Isoquinoline Precursors. Sci. Pharm..

[48] Tumbarski Y., Lincheva V., Petkova N., Nikolova R., Vrancheva R., Ivanov I. (2017). Antimicrobial Activity of Extract from Aerial Parts of Potentilla (*Potentilla reptans* L.). Acad. J. Ind. Technol..

[49] Milusheva M., Todorova M., Gledacheva V., Stefanova I., Feizi-Dehnayebi M., Pencheva M., Nedialkov P., Tumbarski Y., Yanakieva V., Tsoneva S. (2023). Novel Anthranilic Acid Hybrids—An Alternative Weapon against Inflammatory Diseases. Pharmaceuticals.

[50] Milusheva M., Gledacheva V., Stefanova I., Feizi-Dehnayebi M., Mihaylova R., Nedialkov P., Cherneva E., Tumbarski Y., Tsoneva S., Todorova M. (2023). Synthesis, Molecular Docking, and Biological Evaluation of Novel Anthranilic Acid Hybrid and Its Diamides as Antispasmodics. Int. J. Mol. Sci..

[51] Eckhardt S., Brunetto P.S., Gagnon J., Priebe M., Giese B., Fromm K.M. (2013). Nanobio Silver: Its Interactions with Peptides and Bacteria, and Its Uses in Medicine. Chem. Rev..

[52] Frisch M., Trucks G., Schlegel H.B., Scuseria G.E., Robb M.A., Cheeseman J.R., Scalmani G., Barone V., Mennucci B., Petersson G. (2009). Gaussian 09, Revision d. 01.

[53] Lenardon M.D., Sood P., Dorfmueller H.C., Brown A.J.P., Gow N.A.R. (2020). Scalar Nanostructure of the *Candida albicans* Cell Wall; a Molecular, Cellular and Ultrastructural Analysis and Interpretation. Cell Surf..

[54] Yoshimi A., Miyazawa K., Abe K. (2016). Cell Wall Structure and Biogenesis in *Aspergillus* Species. Biosci. Biotechnol. Biochem..

[55] Kircheva N., Dobrev S., Nikolova V., Angelova S., Dudev T. (2022). Theoretical Insight into the Phosphate-Targeted Silver’s Antibacterial Action: Differentiation between Gram (+) and Gram (−) Bacteria. Inorg. Chem..

[56] Cox H., Macquarrie D.A., Simon J.D. (1997). Physical Chemistry: A Molecular Approach Problems and Solutions to Accompany McQuarrie and Simon: Physical Chemistry.

[57] Marenich A.V., Cramer C.J., Truhlar D.G. (2009). Universal Solvation Model Based on Solute Electron Density and on a Continuum Model of the Solvent Defined by the Bulk Dielectric Constant and Atomic Surface Tensions. J. Phys. Chem. B.

[58] Kelly C.P., Cramer C.J., Truhlar D.G. (2005). SM6: A Density Functional Theory Continuum Solvation Model for Calculating Aqueous Solvation Free Energies of Neutrals, Ions, and Solute−Water Clusters. J. Chem. Theory Comput..

[59] Thi M.T.T., Wibowo D., Rehm B.H.A. (2020). Pseudomonas Aeruginosa Biofilms. Int. J. Mol. Sci..

[60] De Oliveira D.M.P., Forde B.M., Kidd T.J., Harris P.N.A., Schembri M.A., Beatson S.A., Paterson D.L., Walker M.J. (2020). Antimicrobial Resistance in ESKAPE Pathogens. Clin. Microbiol. Rev..

[61] Sharma V.K., Yngard R.A., Lin Y. (2009). Silver Nanoparticles: Green Synthesis and Their Antimicrobial Activities. Adv. Colloid. Interface Sci..

[62] Said A., Abu-Elghait M., Atta H.M., Salem S.S. (2023). Antibacterial Activity of Green Synthesized Silver Nanoparticles Using Lawsonia Inermis against Common Pathogens from Urinary Tract Infection. Appl. Biochem. Biotechnol..

[63] Krishnan A., Kandasamy R. (2018). Nonantibiotics Enhance the Antibacterial Activity of Ceftriaxone against Methicillin-Resistant *Staphylococcus aureus*. Asian J. Pharm. Clin. Res..

[64] Krishnan D., Kandasamy R. (2015). Antibacterial Potentiality of Antiulcer and Antispasmodic Drugs with Selected Antibiotics against Methicillin Resistant *Staphylococcus aureus*: In Vitro and in Silico Studies. Bangladesh J. Pharmacol..

[65] Nandini P., Deepnandan D. (2009). Synthetic Process Study and Pharmacological Evaluation of Antispasmodic Drug as Potential Antimicrobial Agent. Asian J. Res. Chem..

[66] Mohamed A., Dayo M., Alahmadi S., Ali S. (2024). Anti-Inflammatory and Antimicrobial Activity of Silver Nanoparticles Green-Synthesized Using Extracts of Different Plants. Nanomaterials.

[67] Habschied K., Krstanović V., Zdunić Z., Babić J., Mastanjević K., Šarić G.K. (2021). Mycotoxins Biocontrol Methods for Healthier Crops and Stored Products. J. Fungi.

[68] Haque M.A., Wang Y., Shen Z., Li X., Saleemi M.K., He C. (2020). Mycotoxin Contamination and Control Strategy in Human, Domestic Animal and Poultry: A Review. Microb. Pathog..

[69] Ramírez-Camejo L.A., Zuluaga-Montero A., Morris V., Rodríguez J.A., Lázaro-Escudero M.T., Bayman P. (2022). Fungal Diversity in Sahara Dust: *Aspergillus sydowii* and Other Opportunistic Pathogens. Aerobiologia.

[70] Seyedmousavi S., Singh K., Srivastava N. (2019). Aspergillosis in Humans and Animals. Recent Trends in Human and Animal Mycology.

[71] Salazar F., Bignell E., Brown G.D., Cook P.C., Warris A. (2021). Pathogenesis of Respiratory Viral and Fungal Coinfections. Clin. Microbiol. Rev..

[72] Kumar P., Kausar M.A., Singh A.B., Singh R. (2021). Biological Contaminants in the Indoor Air Environment and Their Impacts on Human Health. Air Qual. Atmos. Health.

[73] Dacrory S., Hashem A.H., Hasanin M. (2021). Synthesis of Cellulose Based Amino Acid Functionalized Nano-Biocomplex: Characterization, Antifungal Activity, Molecular Docking and Hemocompatibility. Environ. Nanotechnol. Monit. Manag..

[74] Miller A.S., Wilmott R.W. (2019). The Pulmonary Mycoses.

[75] Sugui J.A., Kwon-Chung K.J., Juvvadi P.R., Latgé J.-P., Steinbach W.J. (2015). *Aspergillus Fumigatus* and Related Species. Cold Spring Harb. Perspect. Med..

[76] Tripathi A., Alam A. (2020). Mycotoxins, Mycotoxicosis and Managing Mycotoxin Contamination: A Review. Bio-management of Postharvest Diseases and Mycotoxigenic Fungi.

[77] Hendrickson J.A., Hu C., Aitken S.L., Beyda N. (2019). Antifungal Resistance: A Concerning Trend for the Present and Future. Curr. Infect. Dis. Rep..

[78] Visagie C.M., Houbraken J., Frisvad J.C., Hong S.-B., Klaassen C.H.W., Perrone G., Seifert K.A., Varga J., Yaguchi T., Samson R.A. (2014). Identification and Nomenclature of the Genus *Penicillium*. Stud. Mycol..

[79] Guzmán-Chávez F., Zwahlen R.D., Bovenberg R.A.L., Driessen A.J.M. (2018). Engineering of the Filamentous Fungus *Penicillium chrysogenum* as Cell Factory for Natural Products. Front. Microbiol..

[80] D’Antonio D., Violante B., Farina C., Sacco R., Angelucci D., Masciulli M., Iacone A., Romano F. (1998). Necrotizing Pneumonia Caused by *Penicillium chrysogenum*. J. Clin. Microbiol..

[81] Rios G., Castillo S.B.J., Soto A., De Ajanel M.E.C., Aguilar J., Castellanos J.L.A., Cifuentes J., Conde-Pereira C. (2021). Acute Respiratory Failure with Fatal Outcome due to *Penicillium chrysogenum* in a Man from Guatemala with a Seminoma (Germ Cell Tumor). Chest.

[82] Menezes E.A., Cunha M., Cunha F.A. (2012). Identificação Preliminar de Algumas Espécies Do Gênero *Candida* Spp. Em Meio Cromógeno: Resultados de Dois Anos de Um Estudo Multicêntrico Realizado No Ceará. Rev. Patol. Trop..

[83] Mallmann E.J.J., Cunha F.A., Castro B.N.M.F., Maciel A.M., Menezes E.A., Fechine P.B.A. (2015). Antifungal Activity of Silver Nanoparticles Obtained by Green Synthesis. Rev. Inst. Med. Trop. Sao Paulo.

[84] Hashem A.H., Saied E., Amin B.H., Alotibi F.O., Al-Askar A.A., Arishi A.A., Elkady F.M., Elbahnasawy M.A. (2022). Antifungal Activity of Biosynthesized Silver Nanoparticles (AgNPs) against *Aspergilli* Causing Aspergillosis: Ultrastructure Study. J. Funct. Biomater..

[85] Acharya V.V., Chaudhuri P. (2021). Modalities of Protein Denaturation and Nature of Denaturants. Int. J. Pharm. Sci. Rev. Res..

[86] Venkataesan Kumari B., Mani R., Asokan B.R., Balakrishnan K., Ramasamy A., Parthasarathi R., Kandasamy C., Govindaraj R., Vijayakumar N., Vijayakumar S. (2023). Green Synthesised Silver Nanoparticles Using *Anoectochilus elatus* Leaf Extract: Characterisation and Evaluation of Antioxidant, Anti-Inflammatory, Antidiabetic, and Antimicrobial Activities. J. Compos. Sci..

[87] Faedmaleki F., Shirazi F.H., Salarian A.-A., Ahmadi Ashtiani H., Rastegar H. (2014). Toxicity Effect of Silver Nanoparticles on Mice Liver Primary Cell Culture and HepG_2_ Cell Line. Iran. J. Pharm. Res. IJPR.

[88] Prasannaraj G., Sahi S.V., Ravikumar S., Venkatachalam P. (2016). Enhanced Cytotoxicity of Biomolecules Loaded Metallic Silver Nanoparticles against Human Liver (HepG2) and Prostate (PC3) Cancer Cell Lines. J. Nanosci. Nanotechnol..

[89] Elson C.O., Sartor R.B., Tennyson G.S., Riddell R.H. (1995). Experimental Models of Inflammatory Bowel Disease. Gastroenterology.

[90] Traverso G., Schoellhammer C.M., Schroeder A., Maa R., Lauwers G.Y., Polat B., Anderson D.G., Blankschtein D., Langer R. (2015). Microneedles for Drug Delivery via the Gastrointestinal Tract. J. Pharm. Sci..

[91] Kato S. (2013). Role of Serotonin 5-HT_3_ Receptors in Intestinal Inflammation. Biol. Pharm. Bull..

[92] Yasuda H., Park E., Yun C.-H., Sng N.J., Lucena-Araujo A.R., Yeo W.-L., Huberman M.S., Cohen D.W., Nakayama S., Ishioka K. (2013). Structural, Biochemical and Clinical Characterization of Epidermal Growth Factor Receptor (EGFR) Exon 20 Insertion Mutations in Lung Cancer. Sci. Transl. Med..

[93] Maehara A., Mintz G.S., Shimshak T.M., Ricotta J.J., Ramaiah V., Foster M.T., Davis T.P., Gray W.A. (2015). Intravascular Ultrasound Evaluation of JETSTREAM Atherectomy Removal of Superficial Calcium in Peripheral Arteries. EuroIntervention.

[94] Jiang Y., Zou D., Li Y., Gu S., Dong J., Ma X., Xu S., Wang F., Huang J.H. (2022). Monoamine Neurotransmitters Control Basic Emotions and Affect Major Depressive Disorders. Pharmaceuticals.

[95] Hirota S.A., Helli P.B., Janssen L.J. (2007). Ionic Mechanisms and Ca^2+^ Handling in Airway Smooth Muscle. Eur. Respir. J..

[96] De Francesco E., Sotgia F., Clarke R., Lisanti M., Maggiolini M. (2017). G Protein-Coupled Receptors at the Crossroad between Physiologic and Pathologic Angiogenesis: Old Paradigms and Emerging Concepts. Int. J. Mol. Sci..

[97] Woll K.A., Van Petegem F. (2022). Calcium-Release Channels: Structure and Function of IP_3_ Receptors and Ryanodine Receptors. Physiol. Rev..

[98] Mori M.X., Itsuki K., Hase H., Sawamura S., Kurokawa T., Mori Y., Inoue R. (2015). Dynamics of Receptor-Operated Ca^2+^ Currents through TRPC Channels Controlled via the PI(4,5)P_2_-PLC Signaling Pathway. Front. Pharmacol..

[99] Hill-Eubanks D.C., Werner M.E., Heppner T.J., Nelson M.T. (2011). Calcium Signaling in Smooth Muscle. Cold Spring Harb. Perspect. Biol..

[100] Di Natale M.R., Stebbing M.J., Furness J.B. (2021). Autonomic Neuromuscular Junctions. Auton. Neurosci..

[101] Touyz R.M., Alves-Lopes R., Rios F.J., Camargo L.L., Anagnostopoulou A., Arner A., Montezano A.C. (2018). Vascular Smooth Muscle Contraction in Hypertension. Cardiovasc. Res..

[102] González C., Salazar-García S., Palestino G., Martínez-Cuevas P.P., Ramírez-Lee M.A., Jurado-Manzano B.B., Rosas-Hernández H., Gaytán-Pacheco N., Martel G., Espinosa-Tanguma R. (2011). Effect of 45 Nm Silver Nanoparticles (AgNPs) upon the Smooth Muscle of Rat Trachea: Role of Nitric Oxide. Toxicol. Lett..

[103] Zholos A.V., Bolton T.B., Dresvyannikov A.V., Kustov M.V., Tsvilovskii V.V., Shuba M.F. (2004). Cholinergic Excitation of Smooth Muscles: Multiple Signaling Pathways Linking M2 and M3 Muscarinic Receptors to Cationic Channels. Neurophysiology.

[104] Janssen P., Prins N.H., Meulemans A.L., Lefebvre R.A. (2002). Smooth Muscle 5-HT_2A_ Receptors Mediating Contraction of Porcine Isolated Proximal Stomach Strips. Br. J. Pharmacol..

